# Negative frequency dependent selection on sympatric mtDNA haplotypes in *Drosophila subobscura*

**DOI:** 10.1186/s41065-016-0020-2

**Published:** 2016-11-24

**Authors:** Göran Arnqvist, Zorana Kurbalija Novičić, José A. Castro, Ahmed Sayadi

**Affiliations:** 1 Department of Ecology and Genetics, Animal Ecology, University of Uppsala, Norbyv 18D, SE75236 Uppsala, Sweden; 2 Institute for Biological Research “Siniša Stanković”, University of Belgrade, Despot Stefan Blvd 142, 11000 Belgrade, Serbia; 3 Laboratori de Genètica, Departament de Biologia, Facultat de Ciencies, Edifici Guillem Colom, Universitat de les Illes Balears, Campus de la UIB, Palma de Mallorca, Balears 07122 Spain

**Keywords:** Balancing selection, mtDNA, Life history evolution, Polymorphism, Negative frequency dependent selection, SimuPop

## Abstract

**Background:**

Recent experimental evidence for selection on mitochondrial DNA (mtDNA) has prompted the question as to what processes act to maintain within-population variation in mtDNA. Balancing selection though negative frequency dependent selection (NFDS) among sympatric haplotypes is a possibility, but direct empirical evidence for this is very scarce.

**Findings:**

We extend the previous findings of a multi-generation replicated cage experiment in *Drosophila subobscura*, where mtDNA polymorphism was maintained in a laboratory setting. First, we use a set of Monte Carlo simulations to show that the haplotype frequency dynamics observed are inconsistent with genetic drift alone and most closely match those expected under NFDS. Second, we show that haplotype frequency changes over time were significantly different from those expected under either genetic drift or positive selection but were consistent with those expected under NFSD.

**Conclusions:**

Collectively, our analyses provide novel support for NFDS on mtDNA haplotypes, suggesting that mtDNA polymorphism may at least in part be maintained by balancing selection also in natural populations. We very briefly discuss the possible mechanisms that might be involved.

**Electronic supplementary material:**

The online version of this article (doi:10.1186/s41065-016-0020-2) contains supplementary material, which is available to authorized users.

## Findings

A number of recent experimental studies have shown that alternative naturally occurring mitochondrial DNA (mtDNA) haplotypes encode for alternative phenotypes: mtDNA variation has been shown to be associated with, for example, differences in mitochondrial bioenergetics [1, 2], metabolic rate [3], behavior [4], growth rate [5], life span [6] and fitness [7–9] and these effects are often expressed as epistatic interactions with the nuclear genome [10, 11]. Apart from casting doubt on the dated assumption that mtDNA variation is neutral [12, 13], these findings also raises the important question of what balancing processes act to maintain within-population variation in this haploid, maternally inherited and non-recombining genome in the face of selection where overdominance cannot occur [14]. Theory suggests that mitonuclear epistasis may often contribute to the maintenance of sympatric mtDNA variation, but the conditions under which this is true seem somewhat restricted [15–19]. Gregorius and Ross [20] raised the possibility that negative frequency dependent selection (NFDS) on mtDNA haplotypes, where the relative fitness of a haplotype decreases as its frequency in the population increases, may be responsible for the maintenance of mtDNA polymorphism. Direct experimental evidence for such an effect was lacking until Kazancıoğlu and Arnqvist [21] recently demonstrated that NFDS consistently acts to maintain mtDNA polymorphism in laboratory populations of a seed beetle.

*Drosophila subobscura* is a classic and very interesting model system for the study of mtDNA variation. In this species, variation is ubiquitous and strikingly geographically homogenous, with two dominant mtDNA haplotypes (termed I and II) typically co- occurring within populations at stable frequencies of approximately 50:50, across both the new and the old world [9, 22–28]. Moreover, experimental studies have shown that the two mtDNA haplotypes are not functionally equivalent: flies that harbor these haplotypes differ in major life history traits such as metabolic rate [29], fertility [30], viability, longevity and desiccation resistance [8, 31] and two earlier studies of laboratory cage populations were able to reject genetic drift as the sole mediator of mtDNA haplotype changes [9, 32]. These observations prompted a large experimental effort by Oliver et al. [33], where replicated cage populations of *D. subobscura* were founded by flies carrying either of the two haplotypes (at 50:50) and haplotype frequencies were then monitored for 33 generations. Although genetic drift was rejected in a few of the populations, their analyses were inconclusive because haplotype frequencies remained stable over the course of the experiment and no consistent positive selection favoring either haplotype was detected. Here, we revisit the experiments of Oliver et al. [33], re-analyze their data and significantly extend their findings by showing that they are in fact entirely consistent with NFDS on these sympatric mtDNA haplotypes.

**Fig. 1:**
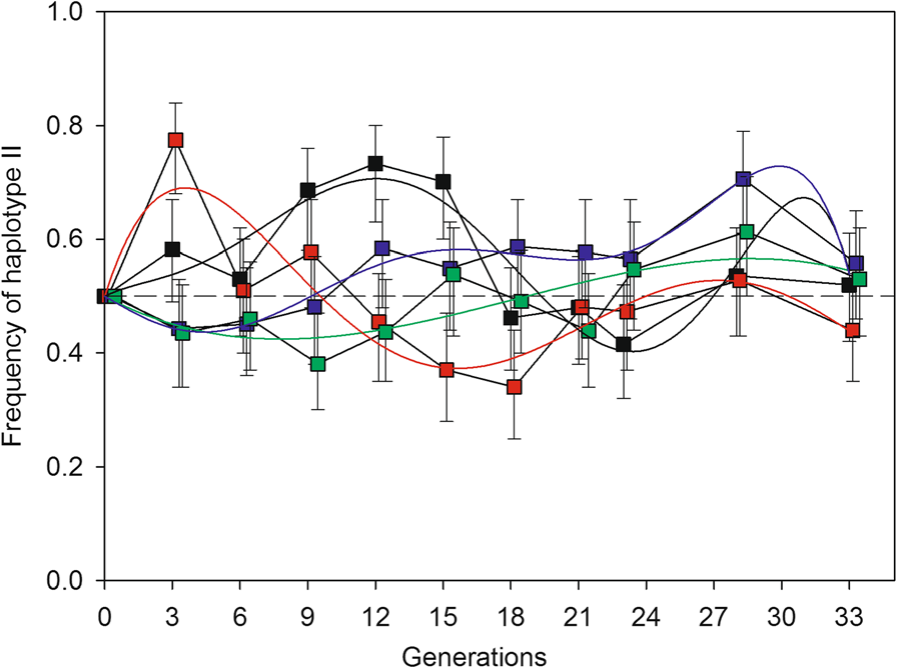
Observed frequency of mtDNA haplotype II in the four replicate cage populations (**a** *black*; **b** *red*; **c** *blue*; **d** *green*) over time. *Error bars* denote empirical 95% CI [46]. Shown are also predicted values from the best fitting polynomial regression models of haplotype frequencies in the four replicates. Data from [33]

For a full account of the experiments, we refer to Oliver et al. [33]. Briefly, four replicate cage populations (A–D) were each founded by exactly 500 flies carrying the type I mtDNA haplotype and 500 carrying type II. These populations were then propagated for 33 subsequent generations, at an average female population size of *N* = 1591 (SD = 679) (Shapiro-Wilk’s test of normality: *P* = 0.229). To track haplotype frequency dynamics, samples of on average 101.7 (SD = 6.2) flies were taken from each population at 10 different points in time (generation 1, 3, 6, 9, 12, 15, 18, 21, 23, 28 and 33) and flies were individually haplotyped. The observed frequency dynamics are illustrated in Fig. 1. PCR screens for cytoplasmic bacteria in these populations were all negative.

To enable quantitative inferences concerning selection on mtDNA haplotypes, we conducted a series of Monte-Carlo (MC) simulations of the populations in Oliver et al. [33]. We used forward-time individual-based simulations in the Python based simulation package simuPOP (version 1.1.1) [34] that mimicked the demography and population dynamics in the population cages (see Additional file 1). Because we were interested in mtDNA dynamics, we modelled a haploid population with strict maternal inheritance of mtDNA haplotypes. Each individual simulation involved four independently evolving populations, representing the four replicate populations, which were evolving under a stable but variable population size. The number of founding females in each population was 500 and the starting frequency of haplotypes was 0.5 for each of the two haplotypes. Population size was thereafter drawn at random from a standard normal distribution (average = 1591, SD = 679) each generation. Selection was applied among adults, such that parents for each offspring were chosen with probabilities that were proportional to their fitness. The relative fitness of each parental individual was a function of its mtDNA haplotypes and contained a fixed additive and a dynamic negative frequency dependent component, as

$$ {W}_I = 1 + {s}_I\mathit{\hbox{--}}\ \left({t}_I{f}_I\right) $$

$$ {W}_{II} = 1 + {s}_{II}\mathit{\hbox{--}}\ \left({t}_{II}{f}_{II}\right) $$

where *W*_*I*_ is the fitness of haplotype I and *W*_*II*_ that of haplotype II, *s* is the additive selection coefficient, *t* a negative frequency dependent selection coefficient and *f* is the prevailing haplotype frequency in any given generation. We then recorded haplotype frequencies in all populations in each of the 10 different points in time where empirical samples were taken. Using the binomial probability distribution with haplotype-specific sampling probabilities set by the observed haplotype frequencies at each time point in each of the simulated populations, we generated 4 × 10 (i.e., 4 populations times the 10 points in time) samples of flies using the actual empirical sample size (average *N* = 101.7 flies per sample) for each simulation. Simulations thus fully incorporated variation within populations over time, across populations and binomial sampling error. These simulated samples were then compared with the observed samples.

We first asked what form of selection maximizes the likelihood of observing the results seen by Oliver et al. [33]. This was achieved in a large number (4 × 10^5^) of MC simulations in which all four selection coefficients (*s*_*I*_, *t*_*I*_, *s*_*II*_ and *t*_*II*_) were independently drawn at random from a standard uniform distribution with a range of 0−2. We assessed model misfit in each MC run as the *χ* ^2^ -value over all 40 samples, treating the empirical samples as the expected and the MC data as the observed haplotype counts. To find the combination of parameter values for the four selection parameters that minimized misfit between the empirical and the MC data, we relied on a full RSM model [35] of log *χ* ^2^ across all MC runs as implemented in SYSTAT^®^ (v. 13.1).

We then tested for NFDS favouring equal haplotype frequencies of the two haplotypes, by asking if the observed haplotype frequencies at the end of the experiment (i.e., generation 33) deviated less from a frequency of 50:50 than would be expected under genetic drift alone. Here, we ran 10^5^ MC simulations in which all four selection coefficients were set to zero and then calculated the *χ* ^2^ -value at generation 33 for the four replicate populations, where the expected counts were based on a 50:50 frequency and the MC data represented the observed haplotype counts. We then asked in what proportion of MC simulations this *χ* ^2^ -value was smaller than or equal to the corresponding empirically observed *χ* ^2^ -value.

Finally, we performed a trend analysis of the empirical data (Fig. 1). The basis of this test is that, under NFDS, positive changes in haplotype frequency over time should tend to be followed by negative changes and vice versa. This is due to the inverse relationship between haplotype frequency and relative fitness under NFDS. Under genetic drift, positive changes should be equally likely to be followed by a negative as a positive change (i.e., a random walk). In contrast, positive selection favouring haplotype II predicts that a positive change in frequency of this haplotype should tend to be followed by another positive change, and vice versa if selection is favouring haplotype I. Thus, transitions where the haplotype frequency change between time *t*-1 to *t* and that between time *t* to *t* + 1 for one of the two haplotypes are of opposite sign,

$$ \mathrm{sign}\ \left(\Delta {f}_{\left[t\mathit{\hbox{-}}1\right]\mathit{\hbox{-}}t}\right)\ne \mathrm{sign}\ \left(\Delta {f_t}_{\mathit{\hbox{-}}\left[t+1\right]}\right) $$

should occur with a probability of *P* = 0.5 under genetic drift, *P* > 0.5 under NFDS and *P* < 0.5 under consistently positive/negative selection. The data of Oliver et al. [33] estimates in total 36 (i.e., 4 × 9) such haplotype frequency transitions and we ask if the distribution of these provides evidence of non-random allele frequency dynamics.

**Fig. 2:**
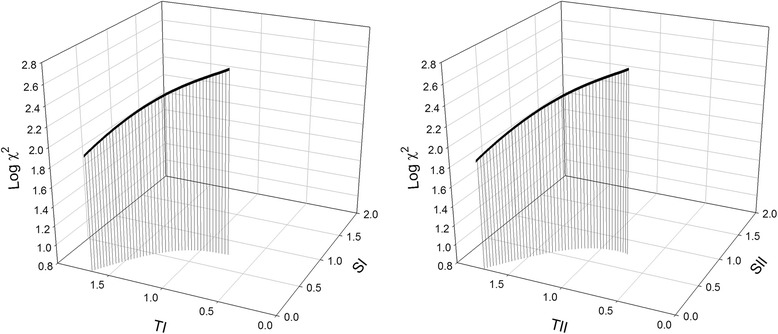
Ridge Analysis of the four-dimensional saddle surface representing the stationary point of a RSM model where the four selection coefficient parameter values minimize the misfit between Monte Carlo simulations and the data observed by Oliver et al. [33]. Here, the X-axis represents negative frequency dependent selection (*T*’s), the Y-axis represents additive selection (*S*’s) and Z-axis represents misfit for haplotype I (*left*) and haplotype II (*right*). The illustrated Ridge Analysis starts off with an arbitrary value of 0.75 for all four selection coefficients, and shows that the best fit with data occurs where there is no additive selection on mtDNA (*S*_*I*_, = *S*_*II*_ = 0) but where negative frequency dependent selection is equally strong on both haplotypes (*T*_*I*_ ≈ *T*_*II*_ > 0)

The full RSM model was highly significant (*P* < 0.001) and explained a very large proportion of the variation in misfit between the empirical data and our MC simulations (*R* ^2^ = 0.69). A canonical analysis of the RSM model showed that the stationary point, where selection coefficient parameter values minimize the misfit between MC simulations and empirical data, was a saddle surface. Ridge Analysis [35] of this four-dimensional surface showed that the combination of selection coefficient parameter values minimizing misfit, thus best mimicking the haplotype frequency dynamics seen, was one in which there was no additive selection on either haplotype (*s*_*I*_, = *s*_*II*_ = 0) but equally strong NFDS on both haplotypes (*t*_*I*_ ≈ *t*_*II*_ > 0) (see Fig. 2).

Our MC simulations also showed that the probability that the haplotype frequencies would deviate as little or less from 50:50 as observed at the end of the experiment, under genetic drift alone, was *P* = 0.039. The corresponding probabilities for the four independent replicate populations were 0.109, 0.399, 0.316 and 0.122 yielding a combined probability of *P* = 0.012 (Stouffer's combined *P*). We thus reject the overall null hypothesis that genetic drift alone is affecting the haplotype frequency dynamics, leaving us with the alternate hypothesis that balancing selection contributes to the stabilization of haplotype frequencies.

Haplotype frequency transitions were of opposite sign 26 out 36 cases (Fig. 1). The binomial probability of observing 26 or more transitions of opposite sign under genetic drift is *P* = 0.006. The corresponding probabilities for the four independent replicate populations were 0.089, 0.089, 0.253 and 0.253 yielding a combined probability of *P* = 0.005 (Stouffer's combined *P*). Hence, we conclude that haplotype frequency transitions of opposite sign were significantly overrepresented in the data, providing more direct support for NFDS. An additional trend analysis, using polynomial regressions forced through a haplotype frequency of 0.5 at time 0, showed that the polynomial model that minimized AIC was of the 6^th^, 5^th^, 6^th^ and 3^rd^ order for replicate A, B, C and D, respectively. This modelling effort, thus, further supported a cyclic dynamic and the inflections points of the fitted functions suggest that the relative fitness of haplotype II becomes notably depressed when reaching a frequency of about *f* = 0.7 and that of haplotype I at about *f* = 0.6 (see Fig. 1).

Our analyses show that the haplotype frequency dynamics observed by Oliver et al. [33] closely match those expected under equally strong NFDS on mtDNA haplotype I and II. Moreover, haplotype frequencies remained significantly more stable than expected under genetic drift and the observed frequency transitions provide evidence for balancing selection through NFSD. Although a few earlier experimental observations are consistent with NFDS on mtDNA in insects [36, 37], our analyses show that the experiments of Oliver et al. [33] are apparently one of very few empirical examples of NFDS on mtDNA haplotypes. Although the experiments of Oliver et al. [33] did not involve starting populations at different haplotype frequencies, as did those of Kazancıoğlu and Arnqvist [21], the analyses presented above collectively provide support for NFDS. We here note that linkage disequilibrium between mtDNA haplotypes and the nuclear genome, e.g. chromosomal inversions, is very low at most in *D. subobscura* [33, 38, 39], which is expected given the fact the two genomes do not cosegregate in males [17, 18].

The study of García-Martínez et al. [32], which also studied the dynamics of the same two mtDNA haplotypes in cage cultures of *D. subobscura*, did not observe the long term maintenance of the two haplotypes seen in the experiments of Oliver et al. [33]. Although the low degree of replication in García-Martínez et al. [32] precludes firm conclusions, it would appear that the two studies differ. One possibility is that differences in experimental design or environmental conditions might have affected the relative fitness of the two haplotypes. Another, perhaps more likely, possibility is that the nuclear genetic background differed between the two studies. The nuclear background is known to, through mitonuclear epistasis, affect the relative fitness of the two haplotypes [29, 31] as well as their evolutionary dynamics [9].

It is currently not possible to assess how widespread NFDS on sympatric mtDNA haplotypes may be or precisely what biological mechanisms may be causing NFDS. With regards to the latter, Lewontin [40] proposed that NFDS within populations may be pervasive because whenever “a genotype is its own worst enemy, its fitness will decrease as it becomes more common” in a manner analogous to similar negative-frequency-dependent processes thought to maintain diversity at a range of ecological and evolutionary scales [41–43]. The necessary and sufficient conditions for this to occur are environmental heterogeneity, genotype-by-environment interactions for fitness and competition for resources within patches. Because mtDNA products contribute to a fundamentally important metabolic cascade, the ATP-producing OXPHOS pathway of the inner mitochondrial membrane, mtDNA haplotype polymorphism should lie at the root of life history trait variation [3, 12, 13]. Individuals carrying alternate sympatric mtDNA haplotypes, essentially representing a non-recombining life history “supergene”, may then show distinct states along multivariate axes of phenotypic variation in life history traits [4]. We suggest that NFDS may be an emergent property of differences in, for example, resource utilization across genotypes related to such life history trait states [43]. Such NFDS may involve selection on mitonuclear genotypes, to the extent that selection can build linkage disequilibrium between mitochondrial and nuclear genes [17, 18]. We note that NFDS on allelic variation in a gene with major life history effects in *D. melanogaster* [44] is apparently generated by differences between genotypes related to juvenile resource utilization under competitive conditions [45]. We suggest that future work should be dedicated to determining the prevalence of NFDS on mtDNA and to unravelling the precise mechanisms though which it occurs.

## Additional file

Additional file 1: SimuPop script. (PDF 23 kb)

## Abbreviations

AIC: Akaike information criterion
CI: Confidence interval
mtDNA: mitochondrial DNA
NFDS: Negative frequency dependent selection
PCR: Polymerase chain reaction
RSM: Response surface method
