# Supplementary material for: Negative frequency dependent selection on sympatric mtDNA haplotypes in Drosophila subobscura
Source: Hereditas. 2016 Nov 24;153:15. doi: 10.1186/s41065-016-0020-2 (PMC5226107; doi:10.1186/s41065-016-0020-2)
Supplement: Additional file 1: — SimuPop script. (PDF 23 kb) [file 41065_2016_20_MOESM1_ESM.pdf]

```
# Script to MC simulate dynamics of the two mtDNA haplotypes
# in Oliver et al's D subobscura cage populations
import simuOpt, os, sys, types, time, random
import simuPOP as sim

start_time = time.time()

# Create new textfile
fileHandle = open ( 'SUB.run.txt', 'w' )
fileHandle.close()

for i in range (1,50):
    print i
    #parameters
    popSize = 500
    generations = 34
    replications = 4

    def demo(gen, pop):
        if gen < 2:
            return 500
        if gen > 1:
            while True:
                loopPopSize = random.normalvariate(1591, 679)
                if (loopPopSize > 398):
                    break
            return [loopPopSize]
    pop = sim.Population(size=popSize, ploidy=1, loci=1, infoFields=['fitness', 'bogus'])

    # Setting selection coefficients as population variables
    pop.dvars().SI = random.uniform(0.0, 0.3)
    pop.dvars().TI = random.uniform(0.0, 0.3)
    pop.dvars().SII = random.uniform(0.0, 0.3)
```

```
    pop.dvars().TII= random.uniform(0.0, 0.3)
    print(pop.vars())
    pop.dvars().I = i

    fileHandle = open ( 'SUB.run.txt', 'a' )
    fileHandle.write ( 'StartRun ' + '\t' + str(i) + '\t' + str(pop.dvars().SI) + '\t' + str(pop.dvars().TI) + '\t' + str(pop.dvars().SII) + '\t' + str(pop.dvars().TII) )
    fileHandle.write ( '\t' )
    fileHandle.close()

    # Initiate replication
    simu = sim.Simulator(pop, rep=4)

    simu.evolve(
        initOps=[
            sim.InitGenotype(freq=[0.5, 0.5])
            ],
        preOps=[
            sim.Stat(alleleFreq=0),
            sim.InfoExec('''fitness = {
            0: 1+SI-(TI*alleleFreq[0][0]),
            1: 1+SII-(TII*alleleFreq[0][1])}[ind.allele(0,)]''',
            exposeInd='ind'),
            sim.Stat(meanOfInfo='fitness'),
        ],
        matingScheme=sim.RandomSelection(subPopSize=demo, ops=sim.CloneGenoTransmitter(infoFields='bogus'), selectionField='fitness'),
        postOps=[
            sim.Stat(alleleFreq=0, at=[3, 6, 9, 12, 15, 18, 21, 23, 28, 33]),
            sim.Stat(popSize=True),
            sim.PyEval(r'''Run %d\t%2d\t%s\t%.3f\t%.3f\t' % (I, gen, subPopSize, alleleFreq[0][0], alleleFreq[0][1])''', at=[3, 6, 9, 12, 15, 18, 21, 23, 28, 33],
output='>>>SUB.run.txt'),
        ],
        gen = generations
    )
```

```
fileHandle = open ( 'SUB.run.txt', 'a' )
fileHandle.write ( '\n' )
fileHandle.close()
```
